# Opening Pandora’s Loot Box: Weak Links Between Gambling and Loot Box Expenditure in China, and Player Opinions on Probability Disclosures and Pity-Timers

**DOI:** 10.1007/s10899-022-10148-0

**Published:** 2022-08-07

**Authors:** Leon Y. Xiao, Tullia C. Fraser, Philip W. S. Newall

**Affiliations:** 1 Center for Digital Play, IT University of Copenhagen, Rued Langgaards Vej 7, 2300 Copenhagen, Denmark; 2 School of Law, Queen Mary University of London, Mile End Road, London, E1 4NS UK; 3 The Honourable Society of Lincoln’s Inn, Lincoln’s Inn, London, WC2A 3TL UK; 4 The City Law School, City, University of London, Northampton Square, Clerkenwell, London, EC1V 0HB UK; 5 Independent Scholar, Edinburgh, UK; 6 School of Psychological Science, University of Bristol, 12a Priory Road, Bristol, BS8 1TU UK; 7 Experimental Gambling Research Laboratory, School of Health, Medical and Applied Sciences, CQUniversity, 400 Kent St, Sydney, NSW 2000 Australia

**Keywords:** Gambling, Video gaming, Loot boxes, Video game regulations, Consumer protection law, Mainland China

## Abstract

Loot boxes are quasi-gambling virtual products in video games that provide randomised rewards of varying value. Previous studies in Western contexts have identified a positive correlation between loot box purchasing and problem gambling severity. A preregistered survey of People’s Republic of China (PRC) video game players (*N* = 879) failed to replicate this correlation. We observed statistically significant but weak positive correlations between loot box expenditure and past-year gambling participation, and between loot box expenditure and impulsiveness. This study *cannot* prove that loot boxes are not disproportionately purchased by people with problem gambling symptomatology in the PRC or that PRC players are not potentially at risk of loot box-related harms. Instead, the evidence suggests that the relationship between loot boxes and gambling might be weaker in the PRC than in Western countries. We identified multiple unique factors about the PRC that might be affecting this relationship. For example, the lotteries are the only legally permitted form of gambling. More gamified electronic gambling products are unavailable. The limited availability of gambling meant that a low level of gambling participation (*n* = 87) was observed, which is a limitation of this study. Additionally, the PRC is presently the only country to legally require loot box probability disclosures as a consumer protection measure. Most loot box purchasers (84.6%) reported seeing loot box probability disclosures, but only 19.3% of this group reported consequently spending less money. Most loot box purchasers (86.9%) thought that pity-timers, which increase the winning probabilities of obtaining rarer rewards, are appropriate for implementation. Future loot box research should give greater consideration to differing cultural contexts and novel consumer protection measures.

## Introduction

Paid loot boxes are monetisation methods in video games that provide the player with randomised rewards of varying in-game and, potentially, real-world value (Drummond et al., 2020b; Nielsen & Grabarczyk, 2019; Xiao et al., 2021b; Xiao, 2022c, 2022d). Loot boxes are prevalent in video games internationally (Rockloff et al., 2020; Xiao et al., 2021a, 2022; Zendle et al., 2020), and are more prevalent in the People’s Republic of China (the PRC)^1^ than in the UK (Xiao et al., 2021c). Loot box purchasing has been observed to be positively correlated with problem gambling in many studies using international samples (Hall et al., 2021; W. Li et al., 2019; Macey & Hamari, 2019; Zendle, 2019b; Zendle et al., 2019a, 2019b; Zendle & Cairns, 2018). Most previous studies have focused on Western countries, including the US (Drummond et al., 2020a; Zendle & Cairns, 2019), Canada (Brooks & Clark, 2019), the UK (Wardle & Zendle, 2021; Zendle, 2019a), Spain (González-Cabrera et al., 2021), Germany (von Meduna et al., 2020), Denmark (Kristiansen & Severin, 2019), Australia (Drummond et al., 2020a; Rockloff et al., 2021) and Aotearoa New Zealand (Drummond et al., 2020a). A secondary analysis (Close et al., 2021) and two meta-analyses (Garea et al., 2021; Spicer et al., 2021) have confirmed this correlation. However, the existing literature is based largely on ‘Western Educated Industrialized Rich and Democratic (WEIRD)’ samples (Henrich et al., 2010). Cultural differences have been identified as a factor that affects gambling behaviours (Ellenbogen et al., 2007; Raylu & Oei, 2004). It is not known whether the same positive correlation can be observed in non-Western countries. Many countries are grappling with how best to regulate loot boxes, including non-Western countries, *e.g.*, Brazil (Dealessandri, 2021), so it is desirable to attempt to replicate this correlation in non-Western countries to broaden the literature and inform forthcoming regulation.

The PRC is the largest video game market in the world (Statista, 2020), and it is the only jurisdiction to uniquely regulate loot boxes by legally requiring video game companies to disclose the probabilities of obtaining loot box rewards as a consumer protection measure (King & Delfabbro, 2019; McCaffrey, 2019; Xiao, 2021a). Only 5.5% of the top-grossing iPhone games with loot boxes chose the most prominent of all possible disclosure formats (automatically displaying the probabilities on the in-game loot box purchase page), suggesting that this consumer protection method could be implemented more effectively (Xiao et al., 2021c). However, it is not known whether players have in fact seen these probability disclosures, and whether they believe that these disclosures have influenced their loot box purchasing behaviour. Obtaining data on these issues can inform the international debate on the effectiveness of probability disclosures as a loot box consumer protection measure (Xiao & Newall, 2022), given that they may be difficult for players to understand and benefit from due to the complexity of loot box reward distribution systems and in-game economies (Ballou et al., 2020).

Additionally, more than half of top-grossing PRC iPhone games include a loot box sub-mechanic, known as a ‘pity-timer’ by the English video gaming community and a ‘保底机制 [guarantee mechanic]’ in Chinese, that causes the probabilities of obtaining rare rewards to change (in almost all cases, to increase) as the player purchases more loot boxes, often up until receiving a rare reward is eventually 100% guaranteed, after which the probabilities are reset (Xiao et al., 2021c). Pity-timers are known to also be implemented in popular video games in Western countries, such as *Hearthstone* (Ballou et al., 2020; Whitson & French, 2021; Xiao & Henderson, 2019). Pity-timers represent a complex aspect of loot box design that players must learn and understand. Pity-timers may also increase the complexity of probability disclosures, because they would need to be regularly updated to reflect the changing probabilities, and hence negatively affect their ability to meaningfully inform players (Xiao & Newall, 2021; Xiao, 2022b). However, it is not known whether players are aware of pity-timers, and what their opinions are on the appropriateness of companies implementing these mechanics.

Unlike in many Western countries, in the PRC, almost all forms of gambling are strictly prohibited by criminal law (People’s Republic of China, The, 2020, art 303). The exceptions are the state-sponsored lotteries (H. Li et al., 2012; Ye et al., 2012; Zeng & Zhang, 2007) and casual wagering between family and friends as entertainment (*e.g.*, card games or Mahjong) (Steinmüller, 2011; Wu & Lau, 2015). Similar to in other parts of the world, illegal gambling in the PRC (i) occurs, and (ii) its scale is difficult to reliably estimate (Bosco et al., 2009; Wu & Lau, 2015). The PRC lotteries’ annual sales from 2016 to 2020 averaged 417.8 billion Chinese Yuan (≈£47.8 billion; US$64.6 billion), equivalent to a per capita spending of 300 Chinese Yuan (≈£34; US$46) (财政部综合司 [Department of General Affairs of the Ministry of Finance], 2021).^2^ This legal spending is lower than in Western countries with more permissive legal gambling markets, which can see annual per capita spending on gambling of roughly ten times that amount (Economist, The, 2017).

Access to and engagement with multiple forms of gambling represent a risk factor for problem gambling in Western countries (Russell et al., 2019). The correlation between loot box purchasing and problem gambling may not appear in the PRC because the lower availability of commercial gambling products may reduce gambling participation, and hence the distribution of problem gambling symptomology (Kesaite & Wardle, 2022; Rose & Day, 1990). Moreover, whilst gambling is associated with perceived social stigma in Western countries (Horch & Hodgins, 2008), negative moral attitudes towards gambling might be even more extreme in non-Western countries (Luk & Bond, 1992; Zeng & Zhang, 2007). Participants from the PRC might be unwilling to disclose personal involvement with perceived stigmatised or illegal activities, perhaps to ‘save face’ (Blaszczynski et al., 1998; Loo et al., 2008; Papineau, 2005), even within an anonymous online survey. The problem gambling prevalence rate in the PRC is not known due to a lack of research (Wu & Lau, 2015). For reference, in Hanguk (South Korea), gambling is less regulated than in the PRC (because sports betting is legal) but viewed with similarly negative moral attitudes. Although Park et al. (2010) reported a problem gambling prevalence rate of 3.0% in South Korea, Williams et al. (2013) reported a rate as low as 0.5%.

The Problem Gambling Severity Index (PGSI) (Ferris & Wynne, 2001) was used by most previous loot box studies to measure problem gambling severity. Certain (if not all) questions of the PGSI are posed with the underlying assumption that the respondent has previously participated in gambling (in the past 12 months, as stated in the introduction to the PGSI) and therefore imply past-year gambling participation in the questions’ wording. To illustrate, the question on ‘loss-chasing’ (‘Have you gone back another day to try to win back the money you lost?’) implies that the respondent must have gambled on a previous occasion and lost on that occasion. A ‘*Never*’ response (which is the only available negative response to this question) fails to differentiate between (a) ‘*Never* participating in gambling’ and (b) ‘*Never* loss-chased.’ Social stigma around gambling participation in the PRC, in particular, may potentially affect participants’ responses. Some non-gamblers (by definition belonging to the former category (a) because they have not participated in gambling at all) may feel uncomfortable with even entering a ‘*Never*’ response because they do not wish to be erroneously perceived as belonging to the latter category (b) and therefore decide to drop out of the survey, if they are forced to complete the PGSI. Other non-gamblers may complete the survey but provide nuisance responses to irrelevant gambling-related questions, *e.g.*, by failing to correctly respond ‘*Never*’ to every single question on the PGSI. Since problem gambling scales are highly skewed, with even most gamblers responding ‘*Never*’ to most questions, nuisance responses might bias studies towards stronger estimates of the correlation between loot box purchasing and problem gambling, particularly if loot box expenditure questions are prone to similar issues.

Finally, impulsiveness is a personality characteristic which commonly correlates with problem gambling (Browne et al., 2019; Secades-Villa et al., 2016). Impulsiveness has also been correlated with loot box purchasing previously (Wardle & Zendle, 2021). A positive correlation between impulsiveness and loot box purchasing in the PRC was therefore hypothesised, as the measurement of this variable would be unaffected by the availability of gambling products or the negative social connotations of gambling in the PRC.

Therefore, the present study sought to examine the correlations between loot box expenditure and problem gambling scores amongst recent gamblers (to attempt to replicate, specifically within this subsample, the positive correlation that has been identified by previous loot box studies amongst the general population more broadly); between loot box expenditure and recent gambling participation status (which might suggest that gamblers more frequently engage with, and spend more money on, loot boxes); and between loot box expenditure and impulsiveness. The following hypotheses were preregistered at https://osf.io/gan6k.

### Hypothesis 1

Loot box expenditure and problem gambling will be positively correlated amongst people who have gambled in the previous 12 months.

### Hypothesis 2

Loot box expenditure will be positively correlated with engagement with gambling in the previous 12 months.

### Hypothesis 3

Loot box expenditure will be positively correlated with impulsiveness.

The present study was also preregistered to describe whether players have ever seen loot box probability disclosures, and whether these disclosures had perceived influences on purchasing behaviour. Finally, as preregistered, the present study describes whether PRC players were aware of pity-timers being implemented, and their opinions on the appropriateness of these mechanics.

## Method

Cross-sectional data were collected in an online survey. The survey was advertised to the general public via Chinese video gaming internet forums, specifically iyingdi.com (旅法师营地), and the Baidu Tieba (百度贴吧) subforums for the mobile game *Arknights* (明日方舟吧) and the Steam video game digital distribution service (steam吧), and five China-related Subreddits (r/China_irl; r/shanghai; r/beijing; r/China; and r/chinalife). We obtained consent to post the survey from moderators prior to publishing. The survey was also circulated through the mailing list of the Chinese regional chapter of DiGRA (Digital Games Research Association) and the First Author’s social media. The advertisements stated that the research would examine how video game players spend money in China. The advertisement and the survey were available in both Simplified Chinese and English and published either in conjunction through bilingual and English channels or exclusively in Simplified Chinese through Chinese channels. The present study was also circulated in expatriate communities (*e.g*., the Subreddit r/chinalife) to collect data from English-language participants resident in the PRC and maximise the number of potential participants. All participants could choose between either language options for the survey. In total, 92.5% of the final sample completed the survey in Chinese. We then presented potential participants with the information sheet (which stated that the survey includes questions about gambling) and asked them to consent to participating. We prevented repeat participation and did not remunerate participants. The present study was approved by the CQUniversity Human Research Ethics Committee (#22774). The survey was open for three months between 14 January and 14 April 2021, as preregistered.

### Participants

In total, 1806 participants initiated the survey. Participants who did not complete the survey (733) and those who did not consent to participating (12) were excluded. The non-completion rate of ~ 40% is similar to Zendle & Cairns’s original loot box study (2018). Participants who reached the final optional comments box were deemed to have completed the survey and were included. Three preregistered exclusion criteria designed to ensure that the sample consisted of adult video game players resident in Mainland China (and not in the Special Administrative Regions of Hong Kong and Macau, where the loot box probability disclosure regulations do not apply and where gambling is regulated, rather than banned) were applied: participants (i) not aged 18 or above (93); (ii) not resident in Mainland China (56); and (iii) who did not play video games in the previous 12 months (15) were excluded. Participants’ IP addresses were not used to estimate their geographic locations in order to exclude non-Chinese Mainland IP addresses, because of the widespread use of virtual private networks (VPNs) to obfuscate one’s own IP address in Mainland China. Prior to data analysis, a small number of participants (18 total) were deemed non-serious and additionally excluded for: including offensive messages in the comments box at the end of the survey (5); reporting their age as being 99 or 125 years (3); reporting having played video games for 111 h or more in the previous week (5); reporting spending 460,000 Chinese Yuan (≈£52,600; US$71,100) or more on video games in the past year (2); reporting spending 300,000 Chinese Yuan (≈ £34,300; US$46,400) on loot boxes in the past year (1); identifying as holding a doctorate degree at 18 years old (1); straight-lining the PGSI with a maximum score of 27 (1). The remaining 879 participants’ responses were retained.

### Measures

#### Demographics

We asked participants to self-report their age, gender, education level, employment status, and income level.

#### Impulsiveness

Impulsiveness was measured using the Barratt Impulsiveness Scale-Brief (BIS-Brief) (Steinberg et al., 2013). Wang et al. (2019) validated a translated Chinese version of the BIS-Brief. We fixed syntax and grammatical issues and then translated the text to Simplified Chinese. We made minor modifications to the vocabulary used to accommodate Mainland Chinese survey participants. Internal reliability of this scale was acceptable (Cronbach’s α = 0.72).

#### Problem Gambling Severity

**Fig. 1:**
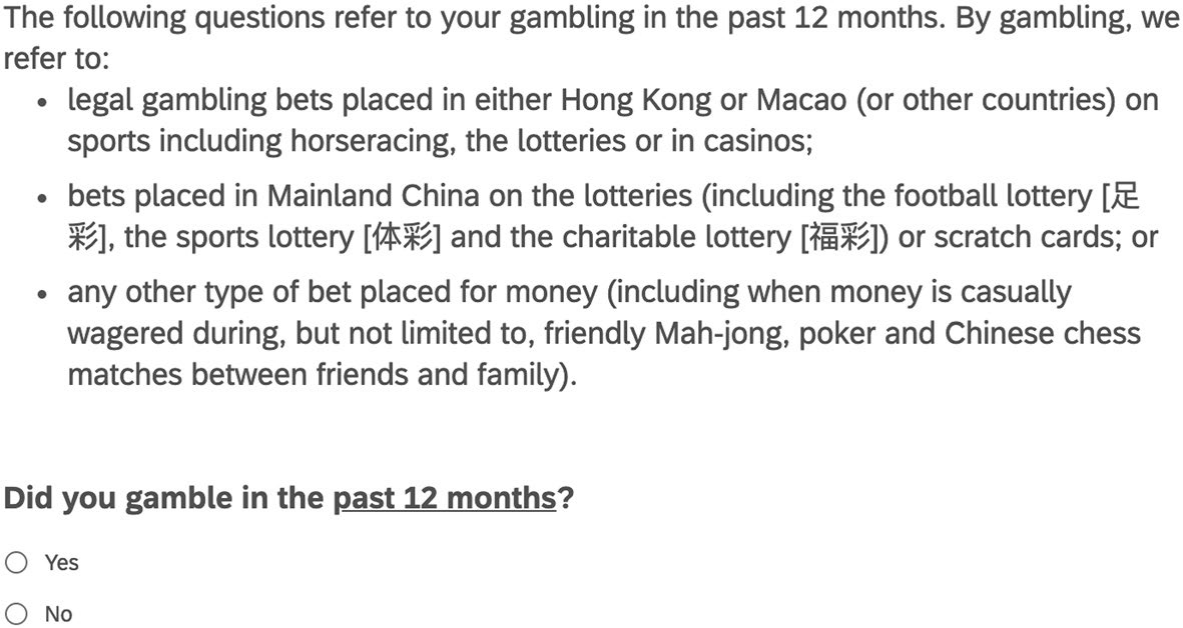
The English version of the definition of ‘gambling’ provided to participants

The Chinese socio-cultural understanding of the term ‘gambling’ (‘赌博’) differs from the international norm (Keovisai & Kim, 2019). To address this, we gave participants a brief introduction to the research team’s ‘Western’ definition of ‘gambling’, as shown in Fig. 1. We then asked participants whether they had gambled in the previous 12 months according to this definition. Participants who answered *no* were skipped to the next question block. Participants who answered *yes* completed the PGSI. The PGSI contains 9 questions relating to gambling behaviour which are each scored on a 4-point Likert scale ranging from 0–3: Never = 0; Sometimes = 1; Most of the time = 2; Almost always = 3. The sum total of the participant’s responses therefore forms a measure of problem gambling severity that ranges from 0–27. The participant was then classified using the revised PGSI scoring system (Currie et al., 2013): 0 = Non-Problem Gambler; 1–4 = Low Risk Gambler; 5–7 = Moderate Risk Gambler; 8 +  = Problem Gambler. Loo et al. (2011) validated a translated Traditional Chinese version of the PGSI. We fixed syntax and grammatical issues and then translated the text to Simplified Chinese. We made minor modifications to the vocabulary used to accommodate Mainland Chinese survey participants. Internal reliability of this scale was good (Cronbach’s α = 0.86).

We screened participants for gambling participation in the previous 12 months and only gave the PGSI to participants who reported participation. This was in contrast to previous studies in the loot box literature, which typically gave gambling scales to all participants. Our methodology was adopted in order to identify the sample’s gambling participation rate, given that an anticipated low gambling participation rate in the PRC was one potential reason why the present study may produce a null result. This approach also follows the methodology of gambling prevalence surveys, which typically only gave gambling scales to participants who report recent gambling participation (Harrison et al., 2020).

#### Loot Box and Video Gaming-Related Questions

We asked participants how many hours of video games they played in the previous week, and how much money they spent on video games in the previous 12 months. We then presented participants with a clear definition for loot boxes and textual examples to avoid misunderstanding and asked them how much money they spent on loot boxes in the previous 12 months, following W. Li et al. (2019).

#### Loot Box Probability Disclosure-Related Questions

We informed participants that the PRC requires video game companies to publish loot box probability disclosures and showed them screenshots of examples. We then asked participants whether they have seen probability disclosures in relation to games they have played, and if a *yes* response was received, we asked whether they had seen these disclosures in-game, on the official website, or elsewhere, and whether their loot box purchasing behaviour has been influenced by any probability disclosures they have seen.

#### Pity-Timer Mechanic-Related Questions

We presented participants with a brief introduction to pity-timer mechanics and example implementations. We then asked participants whether they knew that video game companies implemented pity-timers prior to this survey, and whether they considered it appropriate for companies to implement pity-timers.

### Survey Translation

Except for the two validated measures, whose translation processes were set out above, the survey was created by the First and Last Authors in English, and the back-translation technique was adopted to translate the English survey accurately into Simplified Chinese (Brislin, 1970). The English survey was first translated into Simplified Chinese by the First Author, a bilingual Chinese–English speaker. The Second Author, who was blind to the original English text of the survey and is also a bilingual Chinese–English speaker, then translated the First Author’s Simplified Chinese version of the survey back into English. The First and Second Authors compared the original English and the back-translated English versions and conferred to finalise the Simplified Chinese version of the survey.

Survey materials, raw data and translated Simplified Chinese versions of the BIS-Brief and the PGSI are available via https://osf.io/agbf4/.

### Statistical Tests

We used Spearman’s Rank Correlation tests and a Point Biserial Correlation test for the preregistered hypotheses, and Pearson’s Correlation tests, binomial tests, and a two-sample z-test for proportions during exploratory analyses.

## Results

### Descriptive Statistics: Sample Characteristics

Sample characteristics are shown in Table 1. Participants were predominantly male (709; 80.7%), students (561; 63.8%), and young (*M*_age_ = 23.0, *SD* = 5.9). This is similar to previous studies, which recruited predominantly (~ 90%) males (e.g., Macey & Hamari, 2019; Zendle & Cairns, 2018; Zendle et al., 2019a, 2019b).

**Table 1:** Demographics (N = 879)

Characteristic	Percentage of participants
Age	
18–24	74.5
25–29	15.5
30–34	6.1
35–39	2.3
40–45	1.0
45 +	0.6
Gender	
Male	80.7
Female	14.5
Other	1.0
Prefer not to answer	3.9
Education Level	
Primary School	0.1
Middle School	2.1
High School	6.5
Some university, no degree	51.1
Bachelor’s degree	30.5
Master’s degree	7.7
Doctorate	2.1
Employment Status	
Employed full time (30 or more hours per week)	27.5
Employed part time (less than 30 h per week)	3.3
Unemployed	2.8
Student	63.8
Retired	0.1
Homemaker	0.5
Other	1.9
Income level	
Less than 36,000 Renminbi	63.8
Between 36,000 and 144,000 Renminbi	21.4
Between 144,000 and 300,000 Renminbi	7.7
Between 300,000 and 420,000 Renminbi	3.3
Between 420,000 and 660,000 Renminbi	2.3
Between 660,000 and 960,000 Renminbi	0.8
More than 960,000 Renminbi	0.7
Language option	
Simplified Chinese	92.5
English	7.5

Only a small minority of participants (*n* = 87; 9.9%) self-reported gambling participation in the previous 12 months, with 0.9% of the overall sample meeting the problem gambling score threshold using Currie et al. (2013)’s revised PGSI scoring system (which was adopted by previous loot box studies (e.g., Drummond et al., 2020a; Hall et al., 2021; Zendle & Cairns, 2018, 2019)), as the full breakdown in Table 2 shows.

**Table 2:** Problem gambling severity categories (N = 879)

Problem gambling severity category	Percentage of participants	Percentage of gamblers (*n* = 87)	Loot box expenditure (previous 12 months; Chinese Yuan); Mean (SD)
Non-gamblers	90.1	N/A	1099 (4437)
Non-problem gamblers	5.0	50.6	1211 (3839)
Low risk gamblers	3.5	35.6	1512 (4530)
Moderate risk gamblers	0.5	4.6	775 (932)
Problem Gamblers	0.9	9.2	538 (735)

### Descriptive Statistics: Video Game and Loot Box Engagement

Almost all participants self-reported playing video games in the previous week (851; 96.8%), and spending money on video games in the previous 12 months (755; 85.9%). Just under half of all participants, 428 (48.7%), self-reported purchasing loot boxes in the previous 12 months. The amounts of time and money spent are shown in Table 3.

**Table 3:** Video game and loot box engagement and spending (N = 879)

Characteristic	
Video game expenditure (previous 12 months; Chinese Yuan)	
Mean (SD)	2425 (6447)
95% CI	[1998, 2852]
Minimum–Maximum	0–100,000
Video game time (previous week; hours)	
Mean (SD)	22.4 (18.0)
95% CI	[21.2, 23.6]
Minimum–Maximum	0–100
Loot box expenditure (previous 12 months; Chinese Yuan)	
Mean (SD)	1113 (4379)
95% CI	[823, 1403]
Minimum–Maximum	0–64,800

### Confirmatory Analyses

Hypothesis 1 was tested, for participants who self-reported as gamblers, via the Spearman’s Rank Correlation (which rank-transforms both variables) between loot box expenditure and the summed PGSI score (one-tailed test, *p* = 0.05). A Spearman’s test was used, following previous literature, to reduce the potential impact of outliers on the open-ended loot box expenditure question (e.g., Drummond et al., 2020a; Zendle & Cairns, 2019; Zendle et al., 2019a, 2019b). This test was run because 87 participants (*i.e.*, more than 50 participants) responded that they have gambled in the previous 12 months. We did not find a statistically significant correlation between loot box expenditure and problem gambling (*r*_*s*_(85) = 0.07, *p* = 0.259).

Hypothesis 2 was tested via the Point Biserial Correlation between rank-transformed loot box expenditure and past-year gambling participation (one-tailed test, *p* = 0.05). This test was run because 87 participants (*i.e.*, more than 50 participants) responded that they have gambled in the previous 12 months. Results indicated a statistically significant correlation between loot box expenditure and engagement with gambling in the previous 12 months (*r*_*pb*_(877) = 0.06, *p* = 0.039), although it was very weak.

Hypothesis 3 was tested via the Spearman’s Rank Correlation between rank-transformed loot box expenditure and the summed BIS-Brief score (one-tailed test, *p* = 0.05). Results indicated a statistically significant correlation between loot box expenditure and impulsiveness (*r*_*s*_(877) = 0.06, *p* = 0.038), although it was very weak.

### Descriptive Statistics: Probability Disclosures and Pity-Timers

Overall, 653 of all 879 participants (74.3%) reported seeing loot box probability disclosures, as did 362 of 428 loot box purchasers (84.6%). The locations where participants reported seeing disclosures are detailed in Table 4. A binomial test (H_0_: *p* = 0.5) revealed that participants who indicated that they saw disclosures at either the ‘in-game purchase page only’ or the ‘official website only’ were significantly more likely to have seen disclosures at the more prominent in-game purchase page, rather than at the official website, *p* < 0.001.

**Table 4:** Locations of seen disclosures

	In-game purchase page only	Official website only	Both locations	Other locations only, or other locations and purchase page and/or official website
All participants who saw probability disclosures (n = 653)	311 (47.6%)	73 (11.2%)	216 (33.1%)	53 (8.1%)
Loot box purchasers who saw probability disclosures (n = 362)	168 (46.4%)	37 (10.2%)	127 (35.1%)	30 (8.3%)

Both locations means both on the in-game purchase page and on the official website. Other locations reported by the participants include: social media, including official accounts managed by the video game companies; internet forums; and in-game locations other than the loot box purchase page, such as system notices

As to the perceived effects of seeing probability disclosures, of 362 loot box purchasers who reported seeing disclosures, 262 participants (72.4%) reported that their loot box purchasing behaviour has not been affected by probability disclosures; 70 participants (19.3%) reported buying fewer loot boxes and spending less; and 30 participants (8.3%) reported buying more loot boxes. A binomial test (H_0_: *p* = 0.5) revealed that loot box purchasers who indicated that their purchasing behaviour was influenced were significantly more likely to have bought fewer loot boxes, rather than more loot boxes, *p* < 0.001.

Of all 879 participants, 760 (86.5%) reported having knowledge of pity-timer mechanics before being introduced to the concept in the survey. Of 428 loot box purchasers, 399 (93.2%) reported having prior knowledge of pity-timers. Of all 879 participants, 709 (80.7%) opined that video game companies implementing pity-timer mechanics is either ‘Extremely appropriate’ or ‘Somewhat appropriate.’ Of 428 loot box purchasers, 372 (86.9%) similarly opined that pity-timers are appropriate, as shown in Table 5.

**Table 5:** Opinion on the appropriateness of pity-timers

	Extremely appropriate	Somewhat appropriate	Neither appropriate nor inappropriate	Somewhat inappropriate	Extremely inappropriate
All participants (*N* = 879)	439 (49.9%)	270 (30.7%)	112 (12.7%)	25 (2.8%)	33 (3.8%)
Loot box purchasers (*n* = 428)	240 (56.1%)	132 (30.8%)	44 (10.3%)	8 (1.9%)	4 (0.9%)

### Exploratory Analyses

#### Additional Impulsiveness-Related Analyses

The Pearson’s Correlation between the summed PGSI and BIS-Brief scores was calculated (one-tailed test, *p* = 0.05). Results indicated a statistically significant positive correlation between problem gambling and impulsiveness (*r*(85) = 0.29, *p* = 0.003), replicating previous findings from the gambling literature (Browne et al., 2019; Secades-Villa et al., 2016). The Point Biserial Correlation between the summed BIS-Brief score and past-year gambling participation was calculated (one-tailed test, *p* = 0.05). Results indicated no statistically significant correlation between impulsiveness and past-year gambling participation (*r*_*pb*_(877) = 0.03, *p* = 0.190).

#### Did Participants Quit the Survey at the Gambling-Related Questions?

An analysis of the incomplete responses from the 610 participants who quit the survey during the substantive sections (after completing the consent and screening questions) revealed that only 20 participants (3.3%) quit upon seeing the question asking them to self-disclose past-year gambling participation, or, if an affirmative answer was given to said question, quit during the subsequent PGSI question block. A total of three other question blocks (impulsiveness, demographics, and loot box-related questions) were shown to participants during the substantive sections. A binomial test (H0: *p* = 0.25) revealed that participants were significantly less likely to quit during the gambling question block than during other blocks, *p* < 0.001.

#### Potential Cultural Differences

Of the 813 participants who completed the survey in Simplified Chinese, 73 (9.0%) self-reported as past-year gamblers. In contrast, of the 66 participants who completed the survey in English, 14 (21.2%) self-reported as past-year gamblers. A two-sample z-test for proportions revealed that Chinese-language participants were significantly less likely to be gamblers than English-language participants, *z* = − 3.20, *p* < 0.001. However, exploratory analyses excluding English-language participants showed that neither of the two main confirmatory patterns of significance relating to gambling were affected by the small number of participants who did not complete the survey in Chinese, as shown in Table 6. Notably, results indicated *no* statistically significant correlation between loot box expenditure and impulsiveness (*r*_s_(811) = 0.05, *p* = 0.100) amongst Chinese-language participants, unlike in the overall sample.

**Table 6:** Correlation coefficients for Chinese-language participants (n = 813), one-tailed

	PGSI	Gambled in previous 12 months?	Impulsiveness
Loot box expenditure	*r*_*s*_(71) = .06, *p* = .297	*r*_*pb*_(811) = .07, *p* = .023	*r*_*s*_(811) = .05, *p* = .100
Impulsiveness	*r*(71) = .33, *p* = .002	*r*_*pb*_(811) = .03, *p* = .173	1

#### Including Non-Serious Responses in the Analyses

The criteria for excluding participants deemed non-serious were not preregistered. When the 18 non-serious participants were included in the analyses, none of the confirmatory analyses’ patterns of significance were affected. However, notably, the correlation between impulsiveness and problem gambling severity, which was examined in exploratory analyses in both the overall sample and the Chinese-language participant subsample, became statistically nonsignificant.

## Discussion

Previous Western and international studies have found overall evidence for a positive correlation between loot box purchasing and problem gambling (Close et al., 2021; Garea et al., 2021). However, these correlations may be affected by the availability of gambling in a jurisdiction and cultural factors. The present study was the first to investigate relevant correlations between loot boxes and gambling in the PRC, the largest non-Western and overall video game market. We observed statistically significant but very weak correlations between loot box expenditure and past-year gambling engagement, *r*_*pb*_(877) = 0.06, *p* = 0.039, and between loot box expenditure and impulsiveness, *r*_*s*_(877) = 0.06, *p* = 0.038. The correlation coefficient of *r* = 0.06 for both correlations is considerable less than *r* = 0.2, and therefore the two correlations observed are unlikely to be clinically or practically significant (Ferguson, 2009). Unlike previous studies, the PGSI was only given to participants self-reporting gambling participation in the previous 12 months. The hypothesised correlation between PGSI and loot box expenditure was not observed. However, this may have been due to the low rate of past-year gambling participation (9.9%), which reduced the effective sample size for this test (*n* = 87), meaning that the effect could not have been reliably detected or conclusively rejected by the present study. Caution is therefore urged when interpreting this null result: although it does represent tentative evidence of there being a weaker relationship between loot box purchasing and problem gambling in the PRC than in Western countries, this is not conclusive and requires further replication. Simply put, the present study should *not* be relied upon to argue that this relationship does not exist in the PRC.

In summary, the preregistered analyses suggest a weaker correlation between loot box expenditure and problem gambling in the PRC than has been observed in Western countries (cf. Garea et al., 2021; Spicer et al., 2021). Indeed, the correlation between loot box expenditure and impulsiveness became no longer significant when English-language participants were excluded during exploratory analysis. The inclusion of English-language participants (which the present study was not preregistered to exclude) in the overall sample may therefore have unduly influenced the results and caused the correlations observed to be stronger than the true values amongst PRC players. This might in part be due to English-language and Chinese-language participants having had different video game preferences, which meant that they interacted with different types of loot box mechanics. Exploratory analyses also replicated international evidence on the strength of the correlation between problem gambling and impulsiveness, and provided evidence that participants did not drop out of the survey due to cultural hesitations around responding to gambling-related questions. Finally, as noted above, in this paper, ‘the PRC’ refers to Mainland China exclusively because the applicable gambling laws in Hong Kong, Macau, and Taiwan are different. The sample consisted only of Mainland Chinese residents; therefore, the results should not be overgeneralised to other ethnically Chinese populations.

The past-year gambling participation rate of 9.9% was very low, compared to international rates of 40%–60% (Calado & Griffiths, 2016), as was the estimated problem gambling prevalence rate of 0.9%, compared to an international range of 0.12%–5.8% (Calado & Griffiths, 2016). This is particularly the case given that the sample skewed heavily towards young males—two risk factors for increased problem gambling severity (Browne et al., 2019). All else being equal, a sample largely made up of young males would be expected to have higher gambling participation rates and problem gambling prevalence rates than the international averages. Although exploratory analyses suggest that participants were not dissuaded from responding to gambling-related questions, it remains nonetheless possible that gambling participation (particularly in illegal domestic and remote gambling, and in offshore gambling, although with regards to the latter, the COVID-19 Pandemic rendered travel for leisure purposes during the relevant time difficult, if not impossible, for participants (国家移民管理局 [The National Immigration Administration of the People’s Republic of China], 2020)) may have been underreported due to potential stigmatisation and the criminalisation of gambling in the PRC. Unlike in many Western countries where gambling is legalised, or where gambling is illegal but only gambling organisers are punishable by law, gambling *participation* by individuals is punishable as an imprisonable offence in the PRC, even if the stake is but a relatively modest sum: the Shanghai Police have recognised gambling with stakes just over 100 Chinese Yuan (≈£11.50; US$15.50) as being potentially subject to sanctions in 2017 (Shanghai Municipal Public Security Bureau, 2017). The results presented herein must be interpreted in light of that legal context: if the participants underreported or lied about their gambling participation and engagement, this will likely have weakened the observed relationships and may partially account for why the effect appears to be weaker in the PRC than in previous research in Western countries. Similarly, excessive videogaming participation and engagement might also be stigmatised to a certain degree in the PRC, as demonstrated in part by the legally imposed restrictions on how much time underage players are allowed to spend on online video games (Xiao, 2021b, 2022e). Therefore, the participants might have underreported their videogaming participation and, in particular, loot box expenditure.

One additional possible explanation for the observed muted correlations between loot box expenditure and gambling is that the relatively traditional gambling products available in the PRC may have little appeal to video game players. The lotteries are the only legal commercial gambling products. These do not involve any elements of skill and are pure games of chance, which might therefore be unappealing to players of video games, which are generally games of skill (even though some might involve certain elements of chance). The lotteries may be seen by younger video game players as outdated, unexciting, and unattractive, as the experience involves purchasing physical tickets and waiting for results. In contrast, other gambling products that are legally unavailable in the PRC, such as electronic gambling machines (Schüll, 2012), or equivalent mobile phone casino games (James et al., 2017), are more gamified and have structural characteristics similar to loot boxes, such as ease of use, electronic delivery, and opportunities for rapid play and instant gratification. In support of this explanation, a UK study found that loot box purchasing was more strongly positively correlated with online casino games than with playing bingo or sports betting, and, importantly, was *not* correlated with lottery purchasing (Zendle, 2020). Although the present results appear unsupportive of the loot box purchasing and problem gambling literature (cf. Garea et al., 2021), they could perhaps motivate deeper investigation of this correlation towards the refinement of a more nuanced psychological explanation, *i.e.*, that loot box purchasing is correlated with engagement with and problematic use of specific types of gambling that are gamified and electronic, rather than all types of gambling.

The low gambling participation rate also lends credence to the present study’s design decision to emulate gambling prevalence surveys, rather than previous loot box studies, by screening for gambling participation and not requiring all participants to complete the problem gambling scale. The present study’s methodology can better assist in the understanding of why the present results emerged, and to better utilise these findings, than had the PGSI been given to all participants. For example, any researchers planning a replication loot box study in the PRC or other countries where gambling is prohibited or heavily restricted would be able to plan their sample size requirements based on the knowledge that the reported gambling participation rate is likely to be much lower than in Western contexts.

However, the present design also has a potential shortcoming in that it assumes that the participants who respond negatively to the gambling participation screening question will inevitably respond *0* to all PGSI questions and are non-gamblers. This is not necessarily true: some participants might have responded negatively to the screening question (which was broadly framed) but, had they been pressed to answer the PGSI questions (which the present study did not do), they might contrarily have decided to endorse certain PGSI items when they are forced to think about specific gambling-related situations. Future experimental research should compare the two designs directly to, for example, investigate whether nuisance responding by non-gamblers may have introduced an upwards bias into previous estimates of the loot box purchasing and problem gambling correlation and whether some participants who respond negatively to the gambling participation screening question might actually endorse certain PGSI items due to memory issues and question framing (see Sidloski et al., 2022).

To further contextualise the present findings, that there were only insignificant or muted positive correlations between loot box purchasing and preregistered gambling-related constructs (that the present study managed to assess) in the PRC does not necessarily mean that PRC players are not at risk of being potentially harmed by loot boxes. Gambling represents an outlet for many Western players who exhibit problem gambling-related risk factors. Assuming that PRC players with those same risk factors would also need a similar outlet, due to the illegality of gambling and the unattractiveness of the lotteries, they might decide to (over)spend on loot boxes. Given that the gambling participation rate would be so low in PRC samples, it might be that, due to the country’s restrictive gambling regulatory environment, problem gambling severity is not an appropriate variable for assessing the ‘degree’ of loot box harm amongst PRC players. What was an effective research tool in Western countries appears not to be as useful in the PRC. Further research in the PRC should instead measure harm using different methods and attempt to correlate loot box expenditure with other personality and socio-cultural risk factors associated with the development of problem gambling (such as impulsiveness, which the present study has done and has ruled out) to better understand what kinds of PRC players are more at risk of potential harm.

The present study also investigated players’ opinions of loot box probability disclosures. A previous study suggested that disclosures have been implemented relatively ineffectively by many video game companies, for example, by disclosing probabilities only on the game’s official website and not in-game, which many players may struggle to find (Xiao et al., 2021c). The present results indicate that significantly more players saw probability disclosures in-game, rather than on official websites, despite more games in the PRC choosing to disclose on their official websites than in-game (Xiao et al., 2021c). This demonstrates that in-game disclosures are indeed comparatively more prominent, and are more likely to be seen by players, than official website disclosures. Video game companies should ensure that probability disclosures are prominently published in-game to enable more players to see them. Despite the potential lack of in-game disclosures, most participants in the present study have seen probability disclosures, yet only 19.3% of loot box purchasers who saw disclosures reported buying fewer loot boxes as a consequence. One potential explanation for the apparent ineffectiveness of probability disclosures at reducing spending is that they do not provide enough contextual information and fail to address arguably more pertinent questions that players might have: for example, how much money in total must the player spend to have a competitive account, and would the player need to purchase even more loot boxes in the future even after spending money to obtain the strongest rewards from the currently available type of loot box? The present study’s data are only self-reports, and behavioural measurements of loot box expenditure (*e.g.*, using industry data) could yield different results. However, the results do suggest that stronger interventions, such as increasing the probabilities of winning rare rewards and reducing the total number of potential rewards (Xiao & Newall, 2021), may be needed to effectively reduce potential harms from loot box purchasing. A greater number of customisable and flexible ‘ethical game design’ interventions exist given that loot boxes are purely digital products, in comparison to what is possible in traditional gambling contexts (King & Delfabbro, 2019; Xiao & Henderson, 2019; Xiao & Newall, 2021).

Pity-timer mechanics that change the probabilities of winning rare rewards as more loot boxes are purchased are present in more than half of top-grossing iPhone games available in the PRC (Xiao et al., 2021c). This added element of complexity may invalidate probability disclosures, confuse players, and encourage greater levels of loot box expenditure (King et al., 2019; Whitson & French, 2021; Xiao & Newall, 2021), *i.e.*, be elements of ‘predatory monetisation’ (King & Delfabbro, 2018). However, loot box purchasers were largely aware (93.2%) and supportive (86.9%) of pity-timer mechanics. This may be because nearly all pity-timers objectively *increase* the player’s probabilities of obtaining better rewards, thus seeming to be offering the player a better deal. Indeed, pity-timers may well be welcomed by a majority of low-spending players, but affect high-spending players differently. This pattern of excess harms amongst a high-spending minority has been observed in behavioural data in gamblers (Muggleton et al., 2021). Further research into pity-timers is therefore needed. A qualitative study amongst high-spending players may yield unique insights (Petrovskaya & Zendle, 2021), as supported by this open-ended response from one participant in the present study:

‘Pity-timer mechanics are truly very disgusting. Many times [the valuable reward] is obtained just when the pity [limit] is reached. Sometimes this fundamentally does not conform with the probabilities [disclosed]. [I] truly suspect whether game companies are secretly reducing the probabilities behind the scenes, causing us to draw prizes [i.e., buy loot boxes] not by consulting the probabilities [disclosed], but according to the pity-timer mechanic.’

The present results suggest that caution should be exercised when extrapolating Western findings on new digital markets to other jurisdictions due to cultural and other potential differences. More generally, even within exclusively Western contexts, these results may have opened the metaphorical Pandora’s Box by also beginning to question the underlying theories, methodological choices, and policy proposals that have been widely accepted by the existing loot box literature. More nuanced research focusing on the shared structural characteristics between loot boxes and various specific gambling products, rather than gambling activity broadly defined as a whole, may help to develop more targeted and comprehensive theories on the psychological underpinnings of loot box behaviour. The field would also benefit from reflecting on whether the common and accepted methodological choices could be improved upon, particularly in relation to giving arguably irrelevant gambling-related questions to non-gamblers. Finally, the field should also consider recommending more novel and customised policy options for mitigating any harms from loot boxes, such as fairer and more ethically designed loot boxes (King & Delfabbro, 2019; Xiao & Henderson, 2019; Xiao & Newall, 2021), rather than proposals focused solely on probability disclosures (as advocated for by the video game industry (Entertainment Software Association (ESA), 2019)) or prohibitions on the sale of loot boxes (as adopted in Belgium (Xiao, 2022a) and recommended by certain policymakers in other countries (Select Committee on the Social and Economic Impact of the Gambling Industry of the House of Lords (UK), 2020)). The loot boxes from different games vary greatly, and the amount of money that players might be required to spend in order to be competitive also differs across games. Therefore, the appropriate harm minimisation measures that should be adopted might differ from one game to another. Some games have already changed the design of their loot box reward distribution system and arguably began to adopt certain harm minimisation measures, *e.g.*, Hearthstone (Xiao & Newall, 2021).

The present study has a number of limitations. The sample self-selected into participating in the survey and was not representative: the reported prevalence of gambling participation, problem gambling and loot box purchasing, and average gameplay time and video game spending in the PRC, should not be overgeneralised to other contexts. Additionally, results may have been affected by the locations that the survey was advertised in: for example, the Baidu Tieba subforum for *Arknights*, a game that contains loot boxes and discloses probabilities both in-game and on the game’s official website (Xiao et al., 2021c). Participants originating from this source were naturally more likely to self-report buying loot boxes and seeing probability disclosures, than participants from other more general sources who may only play video games that do not contain loot boxes or do not prominently disclose probabilities.

## Conclusion

The previous literature has shown a positive correlation between loot box purchasing and problem gambling across numerous Western countries (Garea et al., 2021; Spicer et al., 2021). In contrast, the present study observed either insignificant or muted positive correlations between loot box purchasing and preregistered gambling-related constructs in the PRC. This is *not* conclusive proof that loot boxes are not disproportionately purchased by people with problem gambling symptomatology in the PRC or that PRC players are not potentially at risk of loot box-related harms. The present study only presents evidence suggesting that the relationship might be weaker in the PRC than in Western countries. Multiple unique factors about the PRC and about the present study that might have affected this relationship have been identified: the small sample size; gambling participation and engagement being very low (and potentially unreported due to social stigma and gambling’s general illegality); the extremely limited availability of legal gambling products; and certain study design choices that diverged from previous studies. Further replication is needed. Indeed, problem gambling severity may not be an appropriate tool for assessing loot box harms in the PRC and other non-Western countries.

## Data Availability

The data presented in this paper are available via https://osf.io/agbf4/. Survey materials and translated Simplified Chinese versions of the Barratt Impulsiveness Scale-Brief (BIS-Brief) and the Problem Gambling Severity Index (PGSI) are also available at this location.

## Footnotes

1 In this paper, the PRC refers to Mainland China and excludes the Special Administrative Regions of Hong Kong and Macau, and Taiwan, as the applicable laws in these areas are different.

2 Currency conversions were calculated using the currency exchange rates on 29 September 2021.
